# Evaluating Growth and Nitrogen and Phosphorus Removal of Four Microalgae in Different Nutrient Concentrations

**DOI:** 10.3390/biology14091155

**Published:** 2025-09-01

**Authors:** Peizhen Ma, Xiaoqin Li, Biao Wu, Zhihong Liu, Zhuanzhuan Li, Xiujun Sun, Liqing Zhou, Meirong Du

**Affiliations:** 1 State Key Laboratory of Mariculture Biobreeding and Sustainable Goods, Yellow Sea Fisheries Research Institute, Chinese Academy of Fishery Sciences, Qingdao 266071, China; mapz@ysfri.ac.cn (P.M.); dong75190@gmail.com (X.L.); wubiao@ysfri.ac.cn (B.W.); liuzh@ysfri.ac.cn (Z.L.); lizz@ysfri.ac.cn (Z.L.); xjsun@ysfri.ac.cn (X.S.); zhoulq@ysfri.ac.cn (L.Z.); 2 Laboratory for Marine Fisheries Science and Food Production Processes, Qingdao Marine Science and Technology Center, Qingdao 266237, China; 3 Tianjin Key Lab of Aqua-Ecology and Aquaculture, Department of Fishery Science, Tianjin Agricultural University, Tianjin 300384, China

**Keywords:** microalgae, simulated aquaculture wastewater, nitrogen and phosphorus removal, cell growth

## Abstract

The environmental problems brought about by factory-based aquaculture have become increasingly prominent. Reducing nitrogen and phosphorus concentrations in tailwater has become the key to tailwater management. In order to assess the potential of microalgae in removing nitrogen and phosphorus ions from aquaculture wastewater, four microalgae species, i.e., *Chlorella* sp., *Dicrateria zhanjiangensis*, *Nitzschia closterium minutissima*, and *Platymonas subcordiformis*, were used in this study, and their growth and nitrogen and phosphorus removal rates in four nutrient concentrations of simulated aquaculture wastewater were systematically evaluated. After 15 days of cultivation, the cell counts of all four types of microalgae increased. Three species, i.e., *Chlorella* sp., *N. closterium minutissima*, and *P. subcordiformis*, grew best in high PO_4_^3−^ and low NH_4_^+^ medium, whereas *D. zhanjiangensis* possessed best growth in low PO_4_^3−^ and high NH_4_^+^ medium. The removal rate of PO_4_^3−^, NH_4_^+^, NO_3_^−^, and NO_2_^−^ by four microalgae species exceeded 82.64%, 89.06%, 59.27%, and 42.15%, respectively, even though the four microalgae had different performance in the removal of nitrogen and phosphorus. All microalgae in the low-phosphorus groups removed PO_4_^3−^ at significantly lower rates than those in the high–phosphorus groups, while high NH_4_^+^ removal rates were observed in all four microalgae groups. Moreover, in phosphorus-limited conditions, four microalgae exhibit lower removal rates of NO_3_^−^ when nitrogen content was high. The chlorophyll a contents of microalgae in four culture media strictly corresponded to their final cell densities. *P. subcordiformis* exhibited the highest intracellular polysaccharide accumulation in high PO_4_^3−^ and low NH_4_^+^ type medium, whereas *D. zhanjiangensis* demonstrated the strongest protein synthesis capacity in high PO_4_^3−^ and low NH_4_^+^ medium. The activities of acid phosphatase in all microalgae were higher under phosphorus–deficient conditions than phosphorus-sufficient conditions. Our results might provide useful references for microalgae selection in the treatment of different aquaculture wastewater conditions.

## Simple Summary

In aquaculture wastewater treatment, microalgae have shown significant potential in the removal of nitrogen and phosphorus. This study evaluated the growth and nitrogen and phosphorus removal efficiency of four microalgae species—*Chlorella* sp., *Dicrateria zhanjiangensis*, *Nitzschia closterium minutissima,* and *Platymonas subcordiformis*—in simulated aquaculture wastewater with varying nutrient concentrations. The results revealed that four microalgae showed an increase in cell density after 15 days of cultivation, and different microalgae exhibit different abilities to remove nitrogen and phosphorus ions from simulated aquaculture wastewater. *Chlorella* sp., *N. closterium minutissima*, and *P. subcordiformis* grew best in PO_4_^3−^ sufficient and NH_4_^+^ deficient medium, whereas *D. zhanjiangensis* had best growth in PO_4_^3−^ deficient and NH_4_^+^ sufficient medium. In phosphorus-limited conditions, four microalgae exhibited lower removal rates of NO_3_^−^ when nitrogen content was high. The activities of acid phosphatase in all microalgae were higher under phosphorus–deficient conditions than phosphorus-sufficient conditions.

## 1. Introduction

In recent decades, the aquaculture industry has achieved remarkable growth in yield and economic returns. The global aquaculture output reached 130.9 million tons and exceeded fishing output for the first time in 2022 [1]. While the aquaculture scale expands, the environmental problems it causes are increasingly prominent [2,3]. With the improper discharge of aquaculture wastewater and gradual accumulation of wastes, the degradation process of organic matter was overloaded, which significantly changed the physicochemical characteristics of the aquatic environment [4] and ultimately threatened the aquatic ecosystem health [5]. As a result, wastewater treatment technologies have become one of the most important research areas in aquaculture [6,7]. Aquaculture wastewater typically exhibits high concentrations of nitrogen and phosphorus, with higher Chemical Oxygen Demand (COD) and Biological Oxygen Demand (BOD), as well as temperature fluctuations. It is also associated with a notably different microbial community [8,9]. Due to their high nutrient assimilation capacity and CO_2_ fixation efficiency, microalgae have good potential for the treatment of aquaculture wastewater with excessive nitrogen and phosphorus content [10,11].

As the photosynthesizing unicellular microorganisms, microalgae perform biosynthesis and cell proliferation activities through a light energy conversion mechanism [12]. Studies have shown that microalgae can significantly reduce the concentration of inorganic nutrient salts in the environment, e.g., nitrogen and phosphorus, through assimilation during growth, and then synthesize nutrient reserves in the cell [13,14,15]. In the process of nitrogen assimilation, microalgae generally exhibit a preference for ammonium (NH_4_^+^) over nitrate (NO_3_^−^) and nitrite (NO_2_^−^). This preference is primarily due to no need to be reduced and thus the lower energy requirement for assimilating NH_4_^+^ compared to NO3^−^ [16]. NO_2_^−^, while serving as a potential nitrogen source, can inhibit algal growth, likely due to its toxicity and interference with cellular processes [17,18]. For example, NO3^−^ is first catalytically reduced to NO_2_^−^ after being actively transported to the cytoplasm via transmembrane transporter proteins, which is followed by further reduction to NH_4_^+^ catalyzed by nitrite reductase (NIR) [19]. NH_4_^+^ is further involved in the synthesis of intracellular organic matter through glutamine synthetase (GS). Furthermore, the complexity of nitrogen assimilation in microalgae is influenced not only by the nitrogen sources but also by other factors, including the availability of light and carbon sources [20,21,22]. In addition, light intensity, pH, and temperature significantly influence phosphorus absorption during the assimilation process in microalgae. For the phosphorus assimilation, dissolved inorganic phosphate (including forms such as PO_4_^3−^, HPO_4_^2−^, and H_2_PO_4_^−^) is the preferred form of phosphorus assimilated by microalgae [23]. During the early stages of microalgal growth and development, the rate of phosphorus uptake is significantly accelerated due to the involvement of inorganic phosphate in key biological processes such as intracellular energy metabolism, nucleic acid synthesis, and the construction of membrane structures [24]. When phosphorus is in short supply in the environment, phytoplankton maximize the rate of phosphorus acquisition by secreting acid phosphatase (ACP) to meet their requirements for rapid growth and metabolic demands [25]. Research has shown that acidic conditions, high light intensity, and low temperature environments enhance the efficiency of phosphorus uptake [26,27,28,29]. These factors contribute to the intricate nature of the energy dynamics involved in the process.

The concentrations of nitrogen and phosphorus constituents in the wastewater vary due to the aquaculture species exhibiting various bio-metabolic properties, bait composition structure, and culture modes (e.g., indoor factory farming vs. outdoor ponds), such as the concentrations of nitrogen and phosphorus constituents [30]. The NH_4_^+^-N commonly ranges from 0.63 to 5.59 mg/L; NO^3−^-N ranges from 0.38 to 74.80 mg/L; NO_2_^−^-N ranges from 0.13 to 0.17 mg/L; and PO_4_^3−^-P ranges from 0.07 to 6.75 mg/L [31,32,33]. Specifically, in the wastewater from the culture of *Cynoglossus semilaevis* in research conducted by Zheng et al., the concentration of NO_3_^−^ (74.80 mg/L) was approximately twenty times that of PO_4_^3−^ (4.40 mg/L) [34]. But in cultured *tilapia* fish wastewater from a recirculating aquaculture system in West Lafayette, IN, the concentration of PO_4_^3−^ (3.44 mg/L) was much larger than that of NO_3_^−^ (0.35 mg/L), with concentrations of NH_4_^+^ and NO_2_^−^ being 1.90 mg/L and 0.99 mg/L [35]. Given that the forms and concentrations of nitrogen and phosphorus vary widely across different aquaculture systems, the nutrient assimilation capacity of microalgae also shows species-specific differences. Excessively high or low nutrient concentrations often inhibit their growth and productivity [36]. On the other hand, different microalgae may have similar effects in nutrient assimilation. For example, *Chlorella* sp. and *Phaeodactylum tricornutum* could completely remove NH_4_^+^ in simulated marine aquaculture wastewater [37], while *Dicrateria zhanjiangensis* could achieve the same effect in wastewater culturing *Cynoglossus semilaevis* [34]. Of particular note is that using both *Chlorella vulgaris* and *Chlamydomonas reinhardtii* could assimilate NH_4_^+^, NO_3_^−^, and PO_4_^3−^ with all pollutant removal efficiencies of 100% [35]. Therefore, choosing suitable microalgae is ecologically important for more efficient nitrogen and phosphorus removal in wastewater [38].

In this study, we designed four mixed nutrient media with varying concentration gradients of both NH_4_^+^-N and PO_4_^3−^-P and selected four marine microalgae to systematically compare their growth and nitrogen and phosphorus removal abilities. Our aims were: 1: find the growth characteristics of microalgae in different nutrient media; 2: compare the nutrient contents and enzyme activity of microalgae under different media conditions; and 3: evaluate the potential of nitrogen and phosphorus removal of the four microalgae.

## 2. Materials and Methods

### 2.1. Microalgae and Culture Media

Four microalgae species, i.e., *Chlorella* sp., *D. zhanjiangensis*, *Nitzschia closterium minutissima*, and *Platymonas subcordiformis*, were used and cultivated in the Yellow Sea Fisheries Research Institute, Chinese Academy of Fisheries Sciences (Qingdao, China).

The experimental culture media were formulated with the final nutrient concentrations as follows: CuSO_4_·5H_2_O 0.01 mg/L, FeCl_3_·6H_2_O 3.15 mg/L, ZnSO_4_·4H_2_O 0.18 mg/L, MnCl_2_·4H_2_O 0.23 mg/L, Na_2_EDTA 4.36 mg/L, Na_2_MoO_4_·2H_2_O 0.07 mg/L, CoCl_2_·6H_2_O 0.01 mg/L, NaSiO_3_·9H_2_O 20.00 mg/L, Vitamin B_1_ 0.10 mg/L, Vitamin B_12_ 0.10 mg/L, NaNO_3_ 8.10 mg/L, and NaNO_2_ 0.18 mg/L, as well as NH_4_Cl and KH_2_PO_4_ with final concentrations shown in Table 1. All experimental reagents were provided by Sinopharm Chemical Reagent Co., Ltd. (Shanghai, China). Four types of culture media, i.e., LL, LH, HH, and HL, were prepared in this study. The filtered, sterilized seawater used for the media had a pH value of 7.8 ± 0.1 and a salinity value of 34.2 ± 0.1.

### 2.2. Culture Methods

An amount of 100 mL of *Chlorella* sp., *D. zhanjiangensis*, *N. closterium minutissima*, and *P. subcordiformis* in an increased logarithmic phase were centrifuged at 4000 r/min, respectively, and the upper nutrient solution was discarded. The lower layer of each algal species was inoculated into four kinds of media for aseptic culture, with a total volume of 400 mL, and three parallels were set for each treatment group, respectively. During the cultivation process, the ambient temperature was 25 °C, the light intensity was 3500 lx, the light-dark period ratio was 12 h:12 h, and the culture lasted continuously for 15 days. The culture bottle was shaken uniformly every 8 h to avoid adherent cells.

The number of microalgae cells was calculated every 2 days. On days 5, 10, and 15, the concentrations of NH_4_^+^, NO_3_^−^, NO_2_^−^, and PO_4_^3−^ in the culture medium were measured, and the nitrogen and phosphorus removal rate was calculated. After 15 days, the algal liquid was collected and centrifuged at 4000 r/min for 10 min. The algal mud was frozen with liquid nitrogen and then stored at −80 °C for detecting the contents of polysaccharide, protein, and *Chl*a, as well as the enzyme activities of nitrite reductase and acid phosphatase.

### 2.3. Index Measurement and Analysis

#### 2.3.1. Microalgal Biomass Determination

A microplate reader (Infinite F200 PRO, Tecan, Männedorf, Switzerland) was used to measure the optical density (OD) of algal cells, and a flow cytometer (Attune NxT, Thermo Fisher Scientific Inc., Waltham, MA, USA) was used to measure the number of algal cells. The linear relationships between algal cell density (*y*) and OD (*x*) of each microalga were as follows:

*Chlorella* sp.: *y* = 6.0321882 × 10^7^*x* + 1.7829 × 10^4^ (*R*^2^ = 0.9931);

*D. zhanjiangensis*: *y* = 1.6454634 × 10^7^*x* − 1.8398 × 10^4^ (*R*^2^ = 0.9960);

*N. closterium minutissima*: *y* = 1.8399289 × 10^7^*x* − 3.8386 × 10^4^ (*R*^2^ = 0.9923);

*P. subcordiformis*: *y* = 2.095312 × 10^6^*x* + 3.874 × 10^3^ (*R*^2^ = 0.9945).

#### 2.3.2. Nutrient Content

The content of polysaccharide was measured by the Anthrone Colorimetric method [39]. The BCA method was used to measure the total protein content by using the total protein quantitative determination kit (A045–4, Nanjing Jiancheng Bioengineering Institute, Nanjing, China). The content of *Chl*a was measured by the HEE (Hot Ethanol Extraction) method [40], and its concentration was calculated by the following formula:

*C_Chl_*_a_ = 27.9 × [(OD_665_ – OD_750_) – (ODa_665_ – ODa_750_)] × *V*_ethanol_/*V*_sample_

In the formula, *C_Chl_*_a_ represented the concentration of *Chl*a (mg/L); OD_665_ and OD_750_ were the absorbance values at 665 nm and 750 nm wavelength before acidification, respectively; ODa_665_ and ODa_750_ were the absorbance values at 665 nm and 750 nm wavelength after acidification, respectively; *V*_ethanol_ was the final volume (mL) of the extract, and *V*_sample_ was the initial volume (L) of the sample to be measured.

#### 2.3.3. Enzyme Activity

A nitrite reductase kit (R33064, Shanghai Yuanye Biotechnology Company, Shanghai, China) was used to measure the nitrite reductase (NIR) activity of four microalgae. The acid phosphatase (ACP) activities of four microalgae species were measured using an acid phosphatase kit (A060–2-1, Nanjing Jiancheng Bioengineering Institute, Nanjing, China).

#### 2.3.4. Nitrogen and Phosphorus Removal

According to GB/T 12763.4—2007 [41], the NH_4_^+^ concentration was measured by the hypo-bromate oxidimetry method, the NO_3_^−^ concentration was measured by the zinc-cadmium reduction method, the NO_2_^−^ concentration was measured by the diazo-azo method, and the PO_4_^3−^ concentration was measured by the phosphomolybdenum blue spectrophotometry method. The linear relationships between absorbance (*y*) and nutrient concentration (*x*) in each medium were as follows:

NH_4_^+^-N: *y* = 0.0299*x* + 0.0086 (*R*^2^ = 0.9956);

NO_3_^−^-N: *y* = 0.0101*x* + 0.0189 (*R*^2^ = 0.9987);

NO_2_^−^-N: *y* = 0.0307*x* + 0.0179 (*R*^2^ = 0.9987);

PO_4_^3−^-P: *y* = 0.0154*x* + 0.0040 (*R*^2^ = 0.9925).

The nitrogen and phosphorus removal rates were calculated according to the following formula:

$$Re=\frac{So\;-Se}{So}\times 100\%$$

In the formula, *Re* represented the removal rate of NH_4_^+^, NO_3_^−^, NO_2_^−^, or PO_4_^3−^. *So* represented the initial nutrient concentration, and *Se* represented the final nutrient concentration of each ion.

#### 2.3.5. Statistical Analysis

All experimental data were expressed as mean ± standard deviation, with error bars used to express the standard deviation. The SPSS Statistics 27 (IBM SPSS Statistics, New York, NY, USA)was used to conduct one-way ANOVA *t*-test for the groups with different N and P concentrations, and the different significance levels were *p* < 0.05 (significant) and *p* < 0.01 (extremely significant). All figures were drawn using GraphPad prism 9 (GraphPad Software, Boston, MA, USA) and OriginLab Origin 2019 (OriginLab Corporation, Northampton, MA, USA).

## 3. Results

### 3.1. Growth of Chlorella sp. Cultured with Different Nutrient Concentrations and Nitrogen and Phosphorus Removal

**Figure 1:**
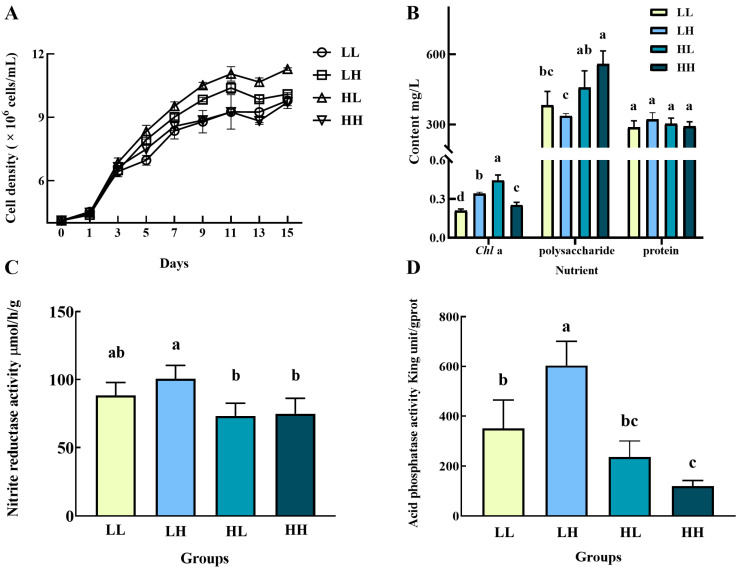
Growth, physiological, and biochemical characteristics of *Chlorella* sp. in four culture media. (**A**) Cell density, (**B**) nutrient content, (**C**) NIR activity, and (**D**) ACP activity. Values marked with different lowercases were significantly different. The LL, LH, HL, and HH groups represent the PO_4_^3−^-deficient and NH_4_^+^-deficient medium, PO_4_^3−^-deficient and NH_4_^+^-sufficient medium, PO_4_^3−^-sufficient and NH_4_^+^-deficient medium, and PO_4_^3−^-sufficient and NH_4_^+^-sufficient medium, respectively. Different lowercase letters indicate significant differences between different groups (Different letters (a, b, c, d) indicate significant differences, while same letters (ab, bc) indicate no significant difference). The same as below.

The cell densities of *Chlorella* sp. in all four media achieved growth in general but fluctuated after day 11 (Figure 1A). A significant difference in cell densities appeared on day 3 between groups LL and HL (*p* < 0.05). The cells in group HL showed a growth advantage throughout the cultivation with a final cell density of 1.1282 × 10^7^ cells/mL, which was a 1.74-fold increase compared to the initial density (0.4113 × 10^7^ cells/mL) and significantly larger than other groups (*p* < 0.05). The groups LL and HH were the lowest two groups after day 5. And the lowest final cell density appeared in group HH (9.707618 × 10^6^ cells/mL), with a 1.36-fold increase compared with the initial cell density. After 15 days, the *Chl*a contents of *Chlorella* sp. cultured under different conditions varied significantly among the four conditions (*p* < 0.05), with the highest value of 0.44 mg/L in group HL and the lowest value of 0.21 mg/L in group HH (Figure 1B). However, the total protein contents were comparable, ranging from 288.04 mg/L to 321.41 mg/L. The group HH had the highest polysaccharide content of 558.69 mg/L, significantly higher than those in groups LL (382.16 mg/L) and LH (336.44 mg/L)(*p* < 0.05). Both the NIR and ACP activities of the *Chlorella* sp. in group LH had the highest value among the four groups (Figure 1C and 1D). The lowest NIR activity value (73.25 μmol/h/g) appeared in group HL, while the lowest ACP activity value (119.83 King unit/gprot) was in group HH. Both the enzyme activities in groups HL and HH had no significant difference (*p* > 0.05).

**Figure 2:**
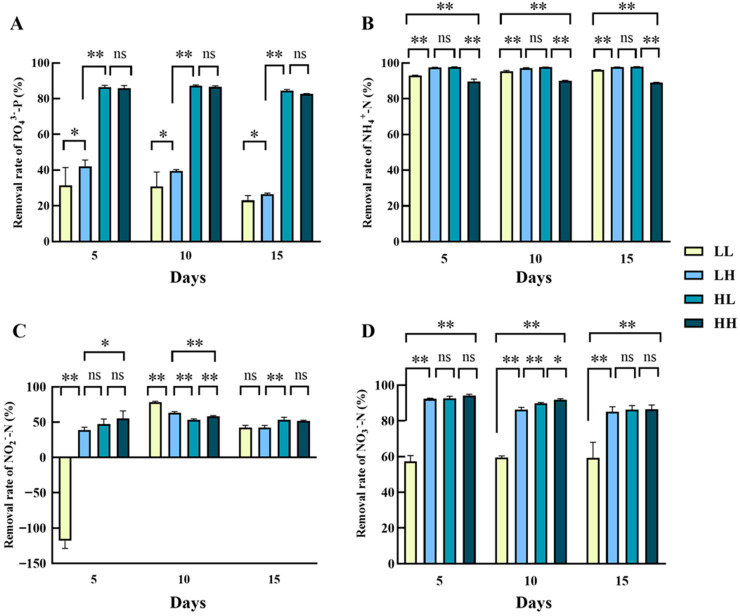
Nitrogen and phosphorus removal rates of *Chlorella* sp. in four culture media. (**A**) PO_4_^3−^removal rate, (**B**) NH_4_^+^removal rate, (**C**) NO_2_^−^removal rate, and (**D**) NO_3_^−^removal rate. Values marked with “*” and “**” were significantly different (*p* < 0.05 and *p* < 0.01, respectively); values marked with ns indicated no significant difference. The same as below.

The PO_4_^3−^ removal rate of *Chlorella* sp. under LL and LH (low phosphorus) culture conditions was significantly lower than the high phosphorus groups on day 5, 10, or 15 (*p* < 0.05) (Figure 2A). There was no significant difference in PO_4_^3−^ removal rate between HL and HH (high phosphorus) groups throughout the cultivation. It is worth noting that on day 15, PO_4_^3−^ removal decreased in all treatment groups to varying degrees compared to day 5. The PO_4_^3−^ removal rates of LH, HL and HH groups on day 15 were all significantly lower than their PO_4_^3−^ removal rates on day 5 and day 10 (*p* < 0.05), and there was no significant difference in the PO_4_^3−^ removal rates of the LL group at the three time points. For the process of NH_4_^+^ removal (Figure 2B), the NH_4_^+^removal rate was significantly lower in the LL group at day 5 than at day 10 and 15 (*p* < 0.05), and there was no significant difference in NH_4_^+^removal between the HL and LH groups at any of the three timings. In all three timings, the relation of the NH_4_^+^ removal among the four groups was HL > LH > LL > HH. By day 15, all four groups achieved more than 89.06% removal rate, with a maximum of 97.86% in all groups. In the removal of NO_2_^−^ by *Chlorella* sp., there were different levels of differences between the groups. The LL group showing negative NO_2_^−^ removal rates on day 5 was −117.94%, indicating net NO_2_^−^ accumulation during the initial incubation (Figure 2C). At day 5, the remaining group was highly significantly more efficient than the LL group in removing NO_2_^−^ (*p* < 0.01). At day 10, the NO_2_^−^ removal rate in group LL increased rapidly to 77.99%, the highest in all groups of NO_2_^−^ removal. The lowest was 53.40% in the HL treatment group. However, at day 15, the NO_2_^−^ removal in the LL treatment group decreased to a minimum of 42.15%. For the removal of NO_3_^−^ (Figure 2D), the LL cultivation conditions (less than 60%) were significantly lower than other groups in the three timings (*p* < 0.01). The NO_3_^−^ removal rates in LH, HL, and HH groups declined compared with those on day 5, with final removal efficiencies of 85.09%, 86.27%, and 86.46%, respectively, by day 15 (*p* < 0.05). The NO_3_^−^ removal rate was significantly higher on day 5 than on day 15, except for the LL group, which had no significant difference in NO_3_^−^ removal at any of the three timings.

### 3.2. Growth of D. zhanjiangensis Cultured with Different Nutrient Concentrations and Nitrogen and Phosphorus Removal

**Figure 3:**
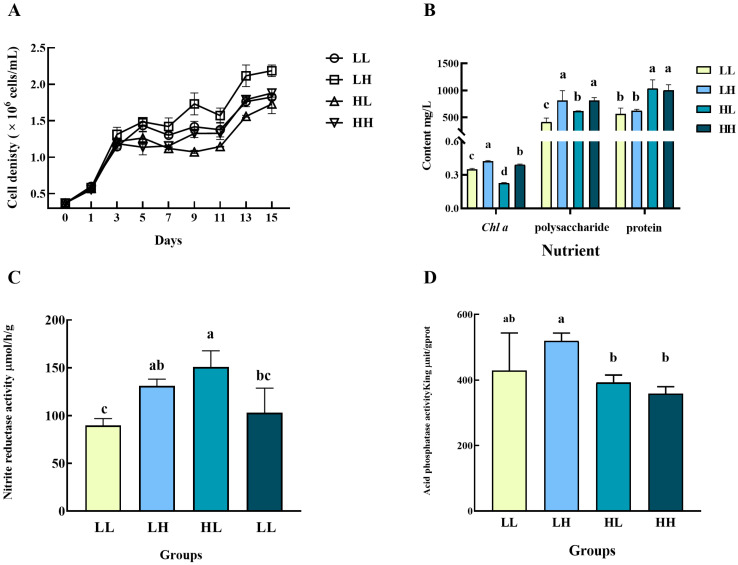
Growth, physiological, and biochemical characteristics of *D. zhanjiangensis* in four culture media. (**A**) Cell density, (**B**) nutrient content, (**C**) NIR activity, and (**D**) ACP activity. Different lowercase letters indicate significant differences between different groups (Different letters (a, b, c, d) indicate significant differences, while same letters (ab, bc) indicate no significant difference.).

As shown in Figure 3A, the cell density of *D. zhanjiangensis* in all groups increased in the beginning but showed a zigzag upward trend after day 3. The microalgal cell proliferation was the fastest under the LH culture conditions, and the cell density was significantly higher than that in all other groups since day 7 (*p* < 0.05). It reached a peak of 2.184666 × 10^6^ cells/mL by the 15 d, increased by 4.90-fold compared with the initial. However, the microalgal cells firstly showed a logarithmic growth under the HL culture conditions, then started to grow negatively after an inflection point on day 5. The final cell density was 1.73 × 10^6^ cells/mL, increased by 3.68-fold compared with the initial density. The *Chl*a content varied under different culture conditions, and that of all groups was significantly different (*p* < 0.05, Figure 3B). The *Chl*a content of *D. zhanjiangensis* was the highest of 0.42 mg/L under the LH condition and the lowest of 0.23 mg/L in the HL group. The *D. zhanjiangensis* under the HL culture condition showed the highest protein content of 1036.81 mg/L, while the LH group showed the best performance of polysaccharide content (813.56 mg/L). Figure 3C shows the NiR activity of *D. zhanjiangensis* in four cultivation environments. The NIR activity in the group HL was the highest of 150.85 μmol/h/g, significantly higher than that in group LL (89.73 μmol/h/g) and HH (102.97 μmol/h/g) (*p* < 0.05). In terms of the ACP activity of *D. zhanjiangensis*, the largest value appeared in group LH (518.86 King unit/gprot), significantly higher than that of the HL (392.35 King unit/gprot) and HH (358.64 King unit/gprot) groups (Figure 3D, *p* < 0.05).

**Figure 4:**
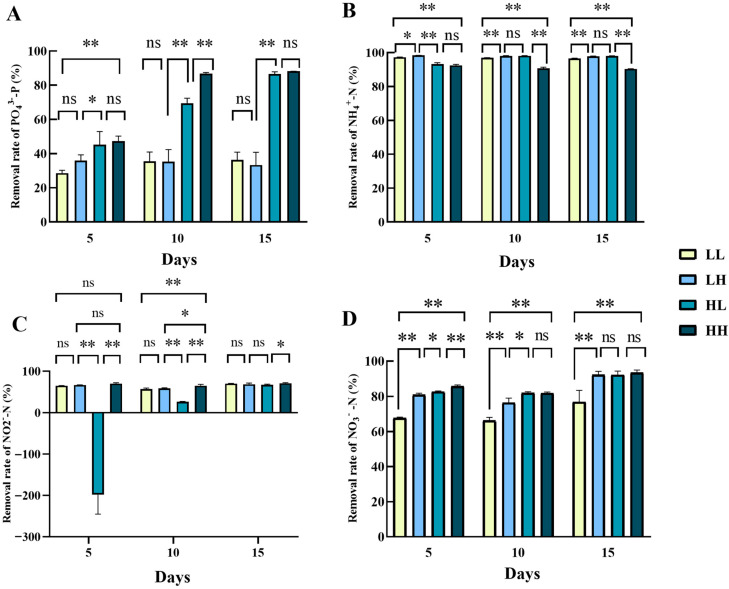
Nitrogen and phosphorus removal rates of *D. zhanjiangensis* in four culture media. (**A**) PO_4_^3−^ removal rate, (**B**) NH_4_^+^ removal rate, (**C**) NO_2_^−^ removal rate, and (**D**) NO_3_^−^ removal rate. Values marked with “*” and “**” were significantly different (*p* < 0.05 and *p* < 0.01, respectively); values marked with ns indicated no significant difference. The same as below.

The PO_4_^3−^ removal efficiencies by *D. zhanjiangensis* varied under different culture conditions, with the group HH being the highest throughout the cultivation (Figure 4A). The two lowest removal efficiency groups were LL and LH, values of which were not significantly different (*p* > 0.05). By day 15, the highest removal rate was in the group HH (88.17%), followed by the HL group of 86.50%. The PO_4_^3−^ removal efficiencies of high PO_4_^3−^ concentration groups were significantly higher than those of low PO_4_^3−^ concentration groups throughout the cultivation (*p* < 0.05). As for the removal of NH_4_^+^, all treatment groups had a high level of removal rate since day 5, and the final removal rate reached more than 90.41% (HH group) at day 15, with a highest value of 98.00% in the group HL (Figure 4B). From day 5 to day 15, the NH_4_^+^ removal rates of three groups, i.e., LL, LH, and HH, showed a decreasing trend, while the HL group increased from day 5 to day 10, but decreased thereafter. Interestingly, similar to that by *Chlorella* sp., the HL culture group of *D. zhanjiangensis* showed an increase instead of a decrease in the concentration of NO_2_^−^ on day 5, and the removal rate was −198.21%, which was extremely significantly lower than other groups (Figure 4C, *p* < 0.01). It gradually increased since then, and by day 15, the NO_2_^−^ removal rate in the HL group amounted to 67.59%, comparable with the LL (70.53%) and LH (68.89%) groups (*p* > 0.01). As shown in Figure 4D, the NO_3_^−^ removal rate of the LL group was always significantly lower than other groups (*p* < 0.01). On day 5, the HH group had the highest NO_3_^−^ removal rates of 85.83% and on day 10, the HL group had the highest value of 82.05%. By day 15, there was no significant difference in the NO_3_^−^ removal rates among the three groups (*p* > 0.05), i.e., LH, HL, and HH, with values of 92.22%, 92.15%, and 93.46%, respectively. It is noteworthy that all treatment groups showed a trend of decrease initially, but then an increase in the process of NO_3_^−^ removal.

### 3.3. Growth of N. Closterium Minutissima Cultured with Different Nutrient Concentrations and Nitrogen and Phosphorus Removal

**Figure 5:**
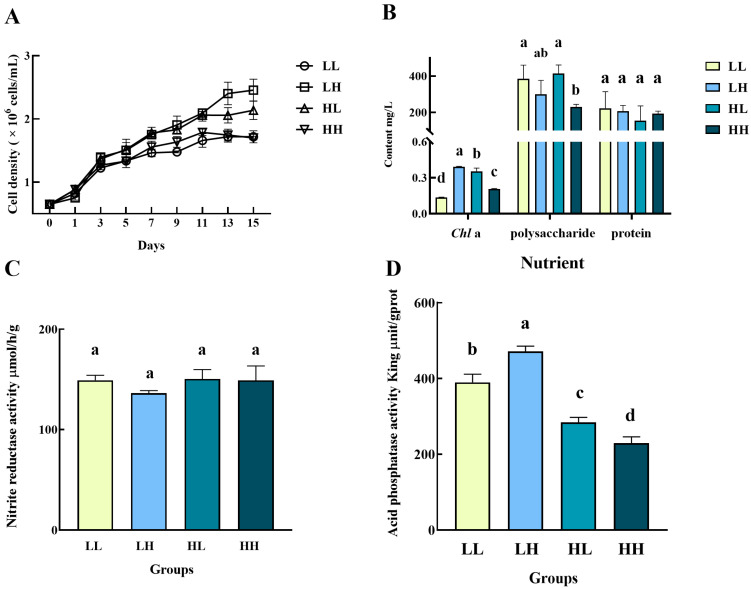
Growth, physiological, and biochemical characteristics of *N. closterium minutissima* in four culture media. (**A**) Cell density, (**B**) nutrient content, (**C**) NIR activity, and (**D**) ACP activity. Different lowercase letters indicate significant differences between different groups (Different letters (a, b, c, d) indicate significant differences, while same letters (ab) indicate no significant difference.).

As shown in Figure 5A, the cell densities of *N. closterium minutissima* showed a continuous growth trend in the LH and HL groups. In contrast, the cell densities in the LL and HH groups experienced a decrease from day 11 to day 13. The cell density in the LH group was comparable with that of the HL group, except on day 13, when there were significantly more cells in the LH group (2.402016 × 10^6^ cells/mL) than in the other groups (*p* < 0.05). The highest final cell density was observed in the LH group, reaching 2.456238 × 10^6^ cells/mL, which was significantly higher than other groups (*p* < 0.05), and increased by 2.78-fold compared with the initial density. In contrast, the final density under the HH condition was the lowest, which was 1.698238 × 10^6^ cells/mL, a 1.61-fold increase compared with the initial density. In terms of the nutrient content, the *Chl*a contents of *N. closterium minutissima* cultured under different conditions varied significantly among the four groups (*p* < 0.05), with the highest value of 0.39 mg/L in group LH and the lowest value of 0.14 mg/L in group LL (Figure 5B). The HL culture group showed the highest accumulation of polysaccharides (414.81 mg/L), while the HH culture group showed the lowest (229.67 mg/L). As for the protein accumulation, the LL group had the highest (222.90 mg/L) while the HL culture group had the lowest (154.62 mg/L). However, the protein contents in all four groups were not significantly different. In addition, there was no significant difference in the level of NIR activities among the four condition groups (Figure 5C). Meanwhile, the ACP activity level of *N. closterium minutissima* cultured in the LH group (471.04 King unit/gprot) was significantly higher than other groups (Figure 5D, *p* < 0.05), followed by the LL type group (389.61 King unit/gprot).

**Figure 6:**
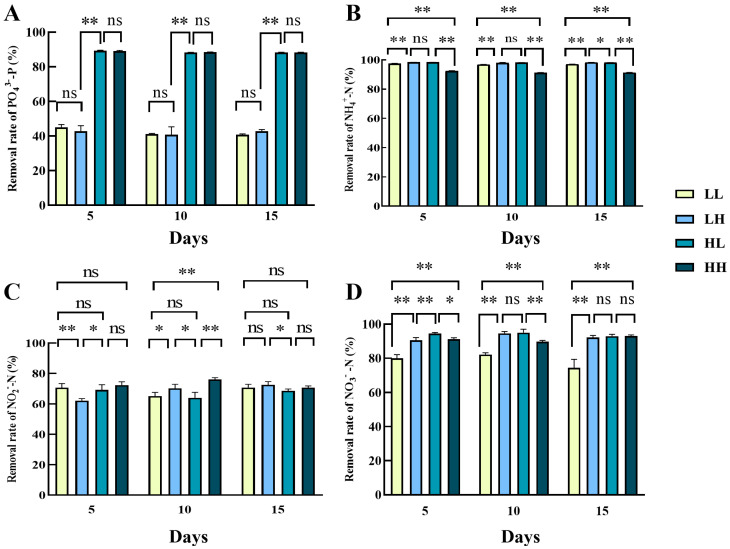
Nitrogen and phosphorus removal rates of *N. closterium minutissima* in four culture media. (**A**) PO_4_^3−^ removal rate, (**B**) NH_4_^+^ removal rate, (**C**) NO_2_^−^ removal rate, and (**D**) NO_3_^−^ removal rate. Values marked with “*” and “**” were significantly different (*p* < 0.05 and *p* < 0.01, respectively); values marked with ns indicated no significant difference. The same as below.

In terms of the nitrogen and phosphorus removal, the PO_4_^3−^ removal rates of *N. closterium minutissima* in low PO_4_^3−^ concentration groups, i.e., LL and LH, were significantly lower than those of the high PO_4_^3−^ concentration groups throughout the cultivation (*p* < 0.05, Figure 6A). However, there was no significant difference in the PO_4_^3−^ removal rate between the same initial PO_4_^3−^ concentration groups. All treatment groups exhibited a high NH_4_^+^ removal rate, ranging from 91.24% to 98.28% on day 15 (Figure 6B), aligning with the situations previously observed in *Chlorella* sp. and *D. zhanjiangensis.* As shown in Figure 6C, the NO_2_^−^ removal rate of *N. closterium minutissima* exhibited distinct trends under the different culture mediums. Specifically, the efficiency initially increased and then decreased in the HH group, whereas in the LL and HL culture media, it shifted from being significantly higher than in LH on day 5 (*p* < 0.05) to lower than in groups LH and HH on day 10. As for the removal of NO_3_^−^, the removal rate under the LL culture medium was always significantly lower than other groups (*p* < 0.01, Figure 6D). The highest NO_3_^−^ removal rate on day 15 was 93.05% under the HH culture condition, whereas the NO_3_^−^ removal rate in group LL was only 74.36%.

### 3.4. Growth of P. Subcordiformis Cultured with Different Nutrient Concentrations and Nitrogen and Phosphorus Removal

**Figure 7:**
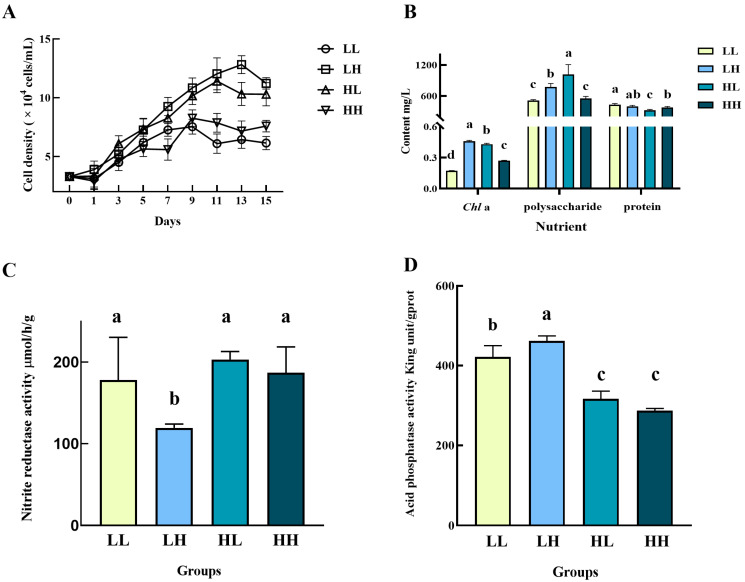
Growth, physiological and biochemical characteristics of *P. subcordiformis* in four culture media. (**A**) Cell density, (**B**) nutrient content, (**C**) NIR activity, and (**D**) ACP activity. Different lowercase letters indicate significant differences between different groups (Different letters (a, b, c, d) indicate significant differences, while same letters (ab) indicate no significant difference.).

The growth of *P. subcordiformis* was similar to that of *N. closterium minutissima*. Although showing an advantage throughout the cultivation and were significantly higher than that in LL and HH medium on day 15 (*p* < 0.05), the cell densities in groups LH and HL experienced from days 11 or 13 (Figure 7A). The highest *P. subcordiformis* cell density on day 15 was 1.12273 × 10^5^ cell/mL under the LH type culture medium, which was a 2.41-fold increase compared to the initial density. The group LL had the lowest final density of only 6.1384 × 10^4^ cell/mL, a 0.87-fold increase compared to the initial density. The results of nutrient determination of *P. subcordiformis* under different culture media were as follows (Figure 7B). The highest *Chl*a content was found in the LH culture medium, which amounted to 0.46 mg/L, while the lowest one was found in the LL group of only 0.14 mg/L. The highest polysaccharide accumulation content was 1013.70 mg/L in the group HL, which was significantly higher than other groups (*p* < 0.05). However, its protein content was the lowest of only 321.74 mg/L, significantly lower than other culture groups (*p* < 0.05). In addition, as shown in Figure 7C, the activities of NiR in the groups LL, HL (the highest, 203.02 μmol/h/g), and HH were comparable, while that in the group LH (119.27 μmol/h/g) was significantly lower than other culture groups (*p* < 0.05). As for the ACP activity, the LH group had the highest value (461.88 King unit/gprot), significantly higher than other groups, followed by the LL group (421.36 King unit/gprot) (*p* < 0.05, Figure 7D).

**Figure 8:**
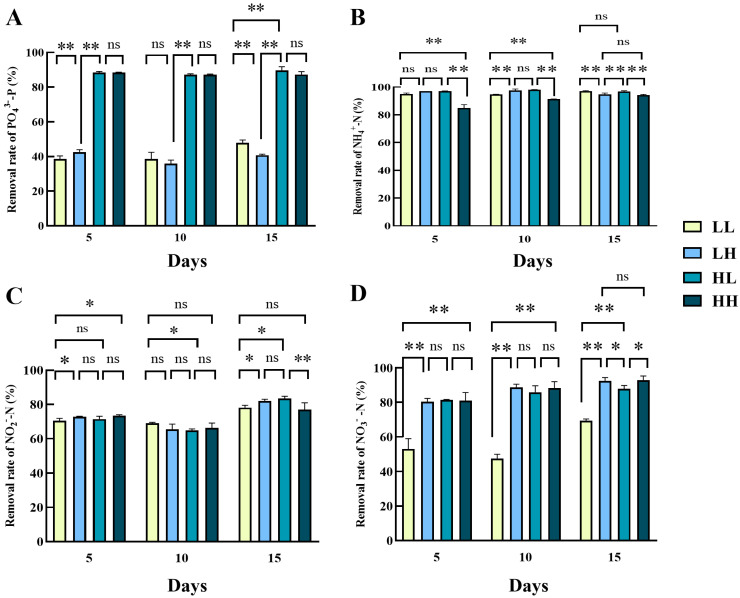
Nitrogen and phosphorus removal rates of *P. subcordiformis* in four culture media. (**A**) PO_4_^3−^ removal rate, (**B**) NH_4_^+^ removal rate, (**C**) NO_2_^−^ removal rate, and (**D**) NO_3_^−^ removal rate. Values marked with “*” and “**” were significantly different (*p* < 0.05 and *p* < 0.01, respectively); values marked with ns indicated no significant difference.

Similar to *Chlorella* sp. and *N. closterium minutissima*, *P. subcordiformis* in the high PO_4_^3−^ concentration groups, i.e., HL and HH, exhibited higher ability in the PO_4_^3−^ removal rates than low PO_4_^3−^ concentration groups throughout the cultivation (*p* < 0.05, Figure 8A). On day 15, the final PO_4_^3−^ removal rates in groups HL and HH were 89.73% and 87.25%, respectively. All the groups exhibited a high NH_4_^+^ removal rate by day 15, peaking at 96.97% in the LL group (Figure 8B). The NH_4_^+^ removal rate of the HH group showed an increasing trend over time, and it always represented the lowest NH_4_^+^ removal rates compared with other groups. Interestingly, the NO_2_^−^ removal rates followed a dip-and-recovery trend across groups, with the HH group showing the lowest rate (74.97%) and the HL group the highest on day 15 (83.56%, Figure 8C). For each group, the NO_2_^−^ removal was significantly higher on day 15 than at the other two timings (*p* < 0.05). The removal rates in the LL group were the lowest among groups over time, which decreased from 52.84% on day 5 to 47.56% on day 10, and then increased to 69.39% on day 15 (significantly lower than other groups, Figure 8D). Notably, the NO_3_^−^removal rates of the LH, HL, and HH groups all increased gradually over time. Eventually, there was no significant difference between the NO_3_^−^ removal rates of the LH (92.34%) and HH (92.77%) groups (*p* > 0.05), while the NO_3_^−^ removal rate of the HL group (87.87%) was significantly lower than the LH and HH groups (*p* < 0.05).

## 4. Discussion

### 4.1. The Growth of Microalgae

Our results showed that different nitrogen/phosphorus (N/P) ratios in simulated aquaculture wastewater directly impacted microalgal growth. The LH type and HL type groups emerged as the top performers in boosting both growth rates and biomass yields. Three microalgae exhibited multiple growth during the first 3 or 5 days across different culture media except *P. subcordiformis*, demonstrating that the initial inorganic nitrogen and phosphorus concentrations in the simulated tailwater effectively supported their proliferation requirements [42,43]. A previous study has shown that the N/P ratios ranging from 6:1 to 18:1 were generally regarded as the optimal range for the growth of microalgae *Scenedesmus obliquus* [44,45]. In contrast, the LL, LH, HL, and HH type mediums contained initial N/P ratios of 9:1, 12:1, 2:1, and 2.5:1 in this study, respectively. These results suggested that the optimal N/P ratio ranges for different microalgae were variable, as the N/P ratios in both HL and HH groups were not within the range for *S. obliquus*. This can also be explained by that in nitrogen and phosphorus-limited conditions, phosphorus stress emerges as the primary limiting factor for cellular proliferation [46,47]. The growth stagnation occurred in the three groups during the later stages. This result corroborates prior research indicating that sustained algal growth is hindered when critical nutrients are exhausted. [48,49]. For *P. subcordiformis*, except for the LH group, the cell densities showed a decline on the first day. It has been demonstrated that the cell wall of *P. subcordiformis* is relatively fragile [50], which could cause a decrease in the number of cells or the short-term stress response to the concentration of nutrients after inoculation [51,52]. It is interesting to find that the points of inflection on the growth curve of *P. subcordiformis* occurred at different times. *P. subcordiformis* utilizes nitrogen and phosphorus from aquaculture wastewater with different efficiency, with 87–95% for nitrogen and 98–99% for phosphorus [53]. The growth of microalgae depends on nutrients, but our results showed that the utilizations were not simply linear. Complex biological reactions related to nitrogen and phosphorus might have happened, such as cell cycle arrest on intermediate metabolism [54,55]. 

Nutrient composition plays an important role in microalgae growth, and different species have varying abilities in the absorption of nitrogen and phosphorus [56,57]. Our findings demonstrate that, except for *D. zhanjiangensis*, the other three microalgae exhibited a trend that when nitrogen or phosphorus was limited, the increase in another nutrient promoted their growth. This may have promoted the synthesis of more nucleic acid and protein by microalgae, thereby synergistically increasing the growth rate [58]. However, when nitrogen or phosphorus was enough, the increase of another nutrient limited their growth. This leads to an imbalance in the N/P ratio within algae cells [59]. This phenomenon is relatively common in ecosystems, indicating that the synergistic effect of nitrogen and phosphorus is not always positive [60,61,62].

### 4.2. Nitrogen and Phosphorus Removal by Microalgae

Microalgae tend to absorb excess phosphorus from the environment during the early stages of culture; surplus phosphorus is stored in vacuoles as polyphosphate and acid polyphosphate, which are then released when phosphorus is scarce to maintain normal cellular metabolism and growth [63]. The ability to absorb phosphorus by microalgae was closely related to the phosphorus concentrations of the environment. Such phosphorus storage mechanisms are well-documented and are vital for sustaining growth under fluctuating nutrient conditions. [64]. Our results showed that all microalgae in the low-phosphorus groups (LL and LH) removed PO_4_^3−^ at significantly lower rates than those in the high–phosphorus groups (HL and HH) (*p* < 0.05), demonstrating all four microalgae have a good removal effect on phosphorus with a concentration of 4.0 mg/L. Li et al. found that in environments with mixed inorganic nitrogen sources, microalgae tend to absorb ammonium nitrogen first and utilize it directly, followed by nitrates and nitrites. [65,66,67]. This explained why the high NH_4_^+^ removal rates were observed in all four microalgae groups. During the initial NO_2_^−^ removal, the concentrations of NO_2_^−^ in both the LL group of *Chlorella* sp. and the HL group of *D. zhanjiangensis* increased by day 5. This result was similar to the previous study [68], where *Chlorella vulgaris* was used to remove NO_2_^−^, indicating that a significant amount of NO_2_^−^ was produced during the early stages of cultivation. *N. closterium minutissima* and *P. subcordiformis* consistently exhibited a high NO_2_^−^ removal rate. In our study, for the removal of NO_3_^−^ in phosphorus-limited conditions, four microalgae exhibited lower removal rates when the nitrogen content was high. However, when phosphorus concentration was elevated, the removal rate of nitrate remained relatively high regardless of the nitrogen level. This suggests that phosphorus content played a limiting role in the efficiency of nitrate removal [69,70]. The previous study [71] demonstrated that diatoms could preferentially use nitrate nitrogen as a nitrogen source in a mixed trophic state environment and for energy assimilation, which might explain why the removal rate of NO_3_^−^ by *N. closterium minutissima* was superior to other microalgae in this study.

### 4.3. Nutrient Contents of Microalgae

Nitrogen deficiency alters microalgal biochemistry, affecting carbohydrates, proteins, pigment profiles, and photosynthesis of microalgae [72,73]. *Chl*a content is a common indicator for evaluating the growth of microalgae [74,75], and in this study, the *Chl*a content of microalgae in four culture media strictly corresponded to the final cell densities. Our results showed that, except for *D. zhanjiangensis*, the other three types of microalgae had the lowest *Chl*a content in LL medium, which was in accordance with the findings of the previous study [76]. Most microalgae tend to synthesize proteins for their biomass accumulation rather than polysaccharides for energy reserves when nitrogen is in sufficient supply [77], and prefer to synthesize reserve polysaccharides when nitrogen becomes scarce [78]. This metabolic shift reflects a common adaptive strategy employed by microalgae when faced with nutrient imbalances. When phosphorus is abundant but nitrogen is lacking, microalgae limit their nucleic acid and ATP synthesis, leading to a decrease in the rate of protein synthesis; alternatively, they shift their products to energy-storing polysaccharides by degrading soluble proteins [79]. This was true in the *Chlorella* sp., *N. closterium minutissima*, and *P. subcordiformis* culture groups in this study. Our findings demonstrate that in the process of polysaccharide accumulation, *P. subcordiformis* exhibited significantly higher content levels than other microalgae species, while *N. closterium minutissima* showed the lowest polysaccharide production. This revealed the distinct interspecific patterns in nutrient storage that likely reflect inherent physiological traits, metabolic pathways, and environmental adaptation mechanisms [80,81].

### 4.4. Enzyme Activities of Microalgae

During the nitrogen assimilation in microalgae, nitrite reductase is used to reduce NO_2_^−^ in the environment and prevent its organisms from being poisoned [82,83]. High activity of NIR usually indicates a higher intracellular nitrogen cycle rate in plants or algae [84,85]. However, since NIR is an intermediate product in the nitrogen metabolism pathway, its activity alone cannot directly reflect the utilization effect of nitrogen. For example, an excessively high concentration of ammonium ions in the environment can also inhibit the activity of NIR [86]. In our study, NIR activity did not exhibit a consistent pattern across the different treatment groups. This represents a limitation of the experiment, primarily due to the assessment being restricted to a single enzyme within the nitrogen assimilation pathway. Under the phosphorus-restricted conditions, microalgae must maintain growth by hydrolyzing intracellular stored phosphorus through extracellular phosphatase to utilize PO_4_^3−^ in organic phosphorus [87]. This allows marine microalgae to cope with nutrient stress even under low phosphorus-limiting conditions [88], and these observations were confirmed in our experiments. Several reports suggest that ACP activity is induced by a combination of nitrogen limitation and phosphorus limitation [89]. In this study, the ACP activity of all microalgae under HL and HH (high phosphorus) groups was lower than LL and LH (low phosphorus) groups, which was consistent with the result that the phosphatase activity of *Emiliania huxleyi* increased in the low phosphorus environment [90].

## 5. Conclusions

Four microalgae had different performance in growth in this study. Three species, i.e., *Chlorella* sp., *N. closterium minutissima*, and *P. subcordiformis*, grew best in high PO_4_^3−^ and low NH_4_^+^ medium, whereas *D. zhanjiangensis* possessed best growth in low PO_4_^3−^ and high NH_4_^+^ medium. Under different concentrations of nutrients, the removal effect of microalgae on nitrogen and phosphorus nutrients varied significantly. *P. subcordiformis* showed the highest removal of both PO_4_^3−^ and NO_2_^−^ in high PO_4_^3−^ and low NH_4_^+^ medium, while *N. closterium minutissima* showed the highest removal of NH_4_^^+^^ in low PO_4_^3−^ and high NH_4_^+^ conditions, and *D. zhanjiangensis* showed the highest removal of NO_3_^−^ in high PO_4_^3−^ and high NH_4_^+^ medium. In contrast, *Chlorella* sp. showed the lowest removal of all nitrogen and phosphorus nutrients. Nutrient accumulation also varied significantly among the four microalgae cultured in different media. *P. subcordiformis* exhibited the highest chlorophyll a content when grown in low PO_4_^3−^ and high NH_4_^+^ medium, whereas intracellular polysaccharide accumulation peaked in high PO_4_^3−^ and low NH_4_^+^-type medium. Notably, *D. zhanjiangensis* demonstrated the strongest protein synthesis capacity in high PO_4_^3−^ and low NH_4_^+^ medium. The nitrogen and phosphorus assimilatory enzyme activities showed distinct variations among different nitrogen–phosphorus mixed culture medium groups. Furthermore, acid phosphatase activity measured under phosphorus-sufficient conditions was consistently lower than phosphorus-deficient conditions. Our research further demonstrated that due to the significant differences in nitrogen and phosphorus concentrations in different aquaculture wastewater environments, it is essential to select appropriate microalgae for wastewater treatment. We have summarized the activities of the four types of microalgae in the simulated aquaculture wastewater in this study (Table 2). These findings provide references for optimizing wastewater remediation and scaling up microalgae production.

## Figures and Tables

**Table 1:** Culture strategies with different ammonium and phosphorus concentrations were used in this study.

NH_4_^+^ Concentration	PO_4_^3−^ Concentration	
	Low (0.8 mg/L)	High (4.0 mg/L)
Low (0.8 mg/L)	LL	HL
High (4.0 mg/L)	LH	HH

**Table 2:** The activities of the four types of microalgae in the simulated aquaculture wastewater.

Characteristics of Aquaculture Wastewater	Simulated Nutrient Concentrations	Microalgae Activities After 15 Day Cultivation							
		Density Increase (Times Larger)				Pollutant Removal Efficiency (%)			
		*Chlorella* sp.	*D. zhanjiangensis*	*N. closterium minutissima*	*P. subcordiformis*	*Chlorella* sp.	*D. zhanjiangensis*	*N. closterium minutissima*	*P. subcordiformis*
PO_4_^3−^ deficient and NH_4_^+^ deficient	PO_4_^3−^: 0.8 mg/L NH_4_^+^: 0.8 mg/L	1.38	3.93	1.64	0.87	PO_4_^3−^: 23.04 NH_4_^+^: 96.13 NO_3_^−^: 59.27 NO_2_^−^: 42.15	PO_4_^3−^: 36.31 NH_4_^+^: 96.64 NO_3_^−^: 76.83 NO_2_^−^: 70.53	PO_4_^3−^: 40.67 NH_4_^+^: 97.07 NO_3_^−^: 74.36 NO_2_^−^: 70.85	PO_4_^3−^: 47.95 NH_4_^+^: 96.97 NO_3_^−^: 69.39 NO_2_^−^: 78.16
PO_4_^3−^ deficient and NH_4_^+^ sufficient	PO_4_^3−^: 0.8 mg/L NH_4_^+^: 4.0 mg/L	1.45	4.90	2.78	2.42	PO_4_^3−^: 26.58 NH_4_^+^: 97.66 NO_3_^−^: 85.09 NO_2_^−^: 42.34	PO_4_^3−^: 33.40 NH_4_^+^: 97.90 NO_3_^−^: 92.22 NO_2_^−^: 68.89	PO_4_^3−^: 42.71 NH_4_^+^: 98.28 NO_3_^−^: 92.32 NO_2_^−^: 72.65	PO_4_^3−^: 40.80 NH_4_^+^: 94.70 NO_3_^−^: 92.34 NO_2_^−^: 81.96
PO_4_^3−^ sufficient and NH_4_^+^ deficient	PO_4_^3−^: 4.0 mg/L NH_4_^+^: 0.8 mg/L	1.74	3.68	2.29	2.13	PO_4_^3−^: 84.5 NH_4_^+^: 97.86 NO_3_^−^: 86.27 NO_2_^−^: 53.16	PO_4_^3−^: 86.50 NH_4_^+^: 98.00 NO_3_^−^: 92.15 NO_2_^−^: 67.59	PO_4_^3−^: 88.20 NH_4_^+^: 98.10 NO_3_^−^: 92.93 NO_2_^−^: 68.69	PO_4_^3−^: 89.73 NH_4_^+^: 96.78 NO_3_^−^: 87.87 NO_2_^−^: 83.56
PO_4_^3−^ sufficient and NH_4_^+^ sufficient	PO_4_^3−^: 4.0 mg/L NH_4_^+^: 4.0 mg/L	1.36	4.08	1.61	1.31	PO_4_^3−^: 82.64 NH_4_^+^: 89.06 NO_3_^−^: 86.46 NO_2_^−^: 51.90	PO_4_^3−^: 88.17 NH_4_^+^: 90.41 NO_3_^−^: 93.46 NO_2_^−^: 71.61	PO_4_^3−^: 88.30 NH_4_^+^: 91.24 NO_3_^−^: 93.05 NO_2_^−^: 70.82	PO_4_^3−^: 87.25 NH_4_^+^: 94.18 NO_3_^−^: 92.77 NO_2_^−^: 76.97

## Data Availability

The original contributions presented in this study are included in the article. Data will be made available upon request.
